# Expression of sphingosine 1-phosphate receptor 4 and sphingosine kinase 1 is associated with outcome in oestrogen receptor-negative breast cancer

**DOI:** 10.1038/bjc.2012.98

**Published:** 2012-03-29

**Authors:** J Ohotski, J S Long, C Orange, B Elsberger, E Mallon, J Doughty, S Pyne, N J Pyne, J Edwards

**Affiliations:** 1 Cell Biology Group, SIPBS, University of Strathclyde, 161 Cathedral St, Glasgow, G4 0RE, UK; 2 Department of Pathology, Glasgow Western Infirmary, Glasgow, G11 6NT, UK; 3 Department of Surgery, Glasgow Western Infirmary, Glasgow, G11 6NT, UK; 4 Unit of Experimental Therapeutics, Institute of Cancer, University of Glasgow, Glasgow, G11 6NT, UK

**Keywords:** sphingosine 1-phosphate, sphingosine kinase, oestrogen receptor, HER2, sphingosine 1-phosphate receptor 4, extracellular signal regulated kinase

## Abstract

**Background:**

We previously reported that sphingosine 1-phosphate receptor 4 (S1P_4_) is expressed and stimulates the ERK-1/2 pathway via a human epidermal growth factor receptor 2 (HER2)-dependent mechanism in oestrogen receptor-negative (ER^−^) MDA-MB-453 breast cancer cells.

**Methods:**

Clinical relevance of S1P_4_ and sphingosine kinase 1 (SK1, which catalyses the formation of S1P) was assessed in a cohort of 140 ER^−^ breast tumours by immunohistochemistry (IHC) and the weighted histoscore method. Additional evidence for a functional interaction between S1P_4_ and SK1 and between HER2 and SK1 was obtained using MDA-MB-453 cells.

**Results:**

High S1P_4_ expression is associated with shorter disease-free (*P*=0.014) and disease-specific survival (*P*=0.004), and was independent on multivariate analysis. In addition, patients with tumours that contain high and low levels of SK1 and S1P_4_, respectively, have a significantly shorter disease-free survival (*P*=0.043) and disease-specific survival (*P*=0.033) compared with patients whose tumours contain both low S1P_4_ and SK1 levels. In addition, high tumour expression of SK1 was significantly associated with shorter disease-specific survival (*P*=0.0001) in patients with HER2-positive tumours. Treatment of MDA-MB-453 cells with the SK1 inhibitor, SKi (2-(p-hydroxyanilino)-4-(p-chlorophenyl)thiazole) reduced the basal and S1P/S1P_4_-induced activation of ERK-1/2 and altered HER2 trafficking in these cells.

**Conclusion:**

These findings highlight an important role for S1P_4_ and SK1 in ER^−^ breast cancer progression.

## 

Effective cancer therapy remains an important unmet medical challenge. For instance, while treatment of oestrogen receptor-positive (ER^+^) breast cancer patients with tamoxifen reduces disease recurrence by 50% at 5 years, patient mortality is only reduced by 28%. Moreover, ER-negative (ER^−^) breast cancer patients have earlier disease recurrence and reduced survival times compared with ER^+^ breast cancer patients. Therefore, there is still a need to identify novel targets for therapeutic intervention that can provide better treatment options. A novel mediator that has an important role in cancer is the bioactive lipid, sphingosine 1-phosphate (S1P; Pyne and Pyne, 2010). Sphingosine 1-phosphate is formed by the phosphorylation of sphingosine to S1P, catalysed by two isoforms of sphingosine kinase (SK), termed SK1 and SK2. Sphingosine 1-phosphate is cleaved by S1P lyase to produce palmitaldehyde and phosphoethanolamine (Pyne and Pyne, 2010). Sphingosine 1-phosphate can also be dephosphorylated by S1P phosphatase to recycle into sphingolipids (Pyne and Pyne, 2010). Sphingosine 1-phosphate is an agonist of sphingosine 1-phosphate (S1P)-specific G-protein-coupled receptors, termed S1P_1-5_ (Pyne and Pyne, 2010) and also binds to intracellular protein targets (Alvarez *et al*, 2010) to promote cell responses, such as growth, migration and survival. Intracellular S1P can also be released from cancer cells and has the potential to bind to S1P receptors to promote cell growth/survival in an autocrine/paracrine manner. This process is called ‘inside-out’ signalling (Takabe *et al*, 2008).

A role for S1P in cancer is evident from studies demonstrating increased expression of SK1 in many different tumour types including breast cancer. Indeed, high tumour expression of SK1 in ER^+^ breast cancer is correlated with poor patient survival rates and earlier disease recurrence on tamoxifen (Long *et al*, 2010a and Watson *et al*, 2010). Sphingosine kinase 1 expression is also higher in ER^–^ compared with ER^+^ breast tumours. Indeed, these patients have a poor prognosis compared with the ER^+^ tumour group (Ruckäberle *et al*, 2008). High expression of SK1 in astrocytoma is also associated with the poor prognosis of patients with Grade 4 tumours (Van Brocklyn *et al*, 2005). Taken together, this evidence strongly supports the notion that SK1 is a ‘sensor’ for promoting cancer growth and survival. Indeed, cancer cells overexpressing SK1 form larger vascularised-resistant tumours compared with tumours lacking ectopically expressed SK1 (Pyne and Pyne, 2010). Sphingosine 1-phosphate receptors are also involved in cancer progression. For instance, S1P binding to S1P_1_ stimulates the migration of fibrosarcoma cells (Fisher *et al*, 2006), whereas binding to S1P_3_ promotes gastric cancer cells migration (Yamashita *et al*, 2006). Furthermore, high S1P_1_ and S1P_3_ expression in ER^+^ breast cancer patients is correlated with poor prognosis (Watson *et al*, 2010).

The human epidermal growth factor receptor 2 (HER2)/*neu*/*c-erbB-2* gene encodes a 185-kDa transmembrane receptor tyrosine kinase. In all, 30% of primary breast cancers contain overexpressed HER2/*neu* and this is correlated with poor prognosis and therapeutic resistance (Borg *et al*, 1990). We have reported that HER2/ErbB2 functionally interacts with sphingosine 1-phosphate receptor 4 (S1P_4_) in ER^−^ MDA-MB-453 breast cancer cells. Sphingosine 1-phosphate binding to S1P_4_ stimulates activation of ERK-1/2 and this is contingent on HER2 (Long *et al*, 2010b). The functional interaction of the S1P_4_ receptor with an oncogene provides evidence that S1P_4_ might have an important role in ER^−^ breast cancer progression. We have therefore analysed tumours from ER^−^ breast cancer patients with respect to the clinical prognostic significance of S1P_4_ and SK1 in these tumours.

## Material and methods

### Materials

All general biochemicals were from Sigma-Aldrich (Poole, UK). High-glucose Dulbecco’s modified Eagle’s medium (DMEM) and European foetal calf serum and penicillin–streptomycin were from Invitrogen (Paisley, UK). Suppliers of antibodies were as follows: anti-phosphorylated ERK-1/2 antibody, Santa Cruz (Santa Cruz, CA, USA); Anti-ERK2 antibody, BD Transduction Laboratories (Oxford, UK); Anti-HER2 antibody, New England Biolabs UK Ltd (Hitchin, UK); Anti-SK1 antibody, Abgent (Abingdon, UK) and kind gift from Dr A Huwiler (University of Berne, Bern, Switzerland); Anti-S1P_4_ antibody, Exalpha (Shirley, MA, USA); anti-*α*-actin antibody and conjugated anti-IgG secondary antibodies, Sigma (Gillingham, Dorset, UK). Sphingosine 1-phosphate was from Avanti Polar Lipids (Alabaster, AL, USA). SKi (2-(*p*-hydroxyanilino)-4-(*p*-chlorophenyl)thiazole) was from Merck Biosciences (Nottingham, UK).

### Patients

All patients (*n*=140) were diagnosed with operable invasive breast carcinoma between 1995 and 1998 in the Greater Glasgow area. These patients received standard adjuvant treatment according to protocols at the time of diagnosis. The patient follow-up details included information on clinical attendances, recurrence and metastasis, date and cause of death as well as adjuvant therapy details. Ethics approval was granted by the Local Ethics Committee.

### Tissue microarray (TMA) construction

Tissue microarrays were already available for use in this study. The 0.6-mm^2^ cores of breast cancer tissue, identified by the pathologist (EM), were removed from representative areas of the tumour taken from breast cancer patients at the time of surgical resection. All TMA blocks were constructed in triplicate.

### Immunohistochemistry

Staining for ER, progesterone receptor (PR), ki67 proliferation index and HER2 had been previously performed for the cohort. The ER and PR protein expression was scored using the weighted histoscore technique. Tumours were classified as ER and PR positive if they had a weighted histoscore of >10. All tumours were classified as ER^−^ and five tumours as PR positive. Ki67 was scored by counting positive and negative nuclei in the tumour specimen and then the percentage of positive cells was calculated to provide a proliferation index. Tumours were classified as having low, moderate or high proliferation index as calculated by tertiles, with 22 tumours being classified as highly proliferative. Human epidermal growth factor receptor 2 expression was scored by the NICE approved scoring system for the HercepTest: 0=no membrane staining; 1+=faint, partial membrane staining; 2+=weak, complete membrane staining in >10% of invasive cancer cells and 3+=intense, complete membrane staining in >10% of invasive cancer cells. A score of 3+ is regarded as positive, 2+ is equivocal and referred for FISH testing and 0 and 1+ are negative (Kajo *et al*, 2007). A total of 39 tumours were classified as HER2 positive.

The TMA slides were first dewaxed and rehydrated through a series of xylene and alcohol washes. Antigen retrieval for SK1 was performed by microwaving the slides under pressure in a citrate buffer for 5 min (pH 6.0) and for S1P_4_ in 10 mM citrate buffer at 96 °C for 20 min. Endogenous peroxidase was blocked using 3% hydrogen peroxide for 20 min and non-specific background staining was reduced for SK1 by blocking with casein for 20 min and for S1P_4_ by blocking with 1 : 20 horse serum for 30 min. The sections were then incubated with the primary antibody (1 : 50 SK1, and 1 : 100 S1P_4_) at 25 °C in a humidified incubator for 60 min (SK1) and 4 °C overnight (S1P_4_). EnVision-HRP conjugate (DAKO, Cambridgeshire, UK) was used for signal amplification, and positive staining was identified using 3,3′-diaminobenzidine chromagen (Vector Laboratories, Peterborough, UK). The slides were then counterstained with haematoxylin and Scott’s Tap Water Substitute before dehydration and mounting.

### Scoring

The protein expression of each core (three per tumour specimen) was assessed using the weighted histoscore method (H score method). The weighted histoscore grades staining intensity as negative (0), weak (1), moderate (2) and strong (3) and then multiplies the percentage of tumour cells within each category. The histoscore range is from 0 (minimum) to 300 (maximum). Agreement between observers was calculated using interclass correlation coefficient.

### Statistical analysis

Disease-specific survival rates were generated using the Kaplan–Meier method. The log-rank test was used to compare significant differences between subgroups using univariate analysis. On the basis of the results of the univariate analysis a multivariate analysis was then carried out. The multivariate stepwise Cox-regression analysis was performed to identify factors that were independently associated with disease-specific death. A stepwise backward procedure was used to derive a final model of the variables that had a significant independent relationship with survival. To remove a variable from the model, the corresponding *P*-value had to be >0.05.

Inter-relationships between clinical parameters, PR, HER2, SK1, S1P_4_ and Ki67 status were calculated using the *χ*^2^ test. Data are expressed as median and range. The statistical analyses were performed using a statistical software package (SPSS 15.0 Inc., Chicago, IL, USA).

### Cell culture

MDA-MB-453 breast cancer cells were obtained from the ATCC (Rockville, MD, USA) and grown in a monolayer culture in high-glucose DMEM with 10% European Fetal Calf Serum (EFCS) and 1% pen–strep (penicillin G sodium 10^4^ units ml^−1^–streptomycin sulphate 10 mg ml^−1^) at 37 °C with 5% CO_2_. Cells were serum starved for 48 h before stimulation with S1P.

### Western blotting

Analysis of proteins by SDS–PAGE and western blotting was performed as described previously by us (Alderton *et al*, 2001) using anti-phosphorylated ERK-1/2, anti-*α*-actin, anti-SK1 and anti-ERK2 antibodies.

### Immunofluorescence microscopy

Cells were plated onto autoclaved 13-mm glass coverslips and grown to 60% confluence before serum starvation for 48 h before stimulation. Cells were fixed with 3.7% formaldehyde in phosphate-buffered saline (PBS) for 10 min, permeabilised with 0.1% Triton X-100 in PBS for 1 min before incubation in blocking solution (5% EFCS and 1% bovine serum albumin in PBS) for 30 min at room temperature. Coverslips were then incubated with anti-HER2 antibody (1 : 100 dilution in blocking solution) overnight at 4 °C. Coverslips were washed with PBS and incubated with FITC-conjugated anti-rabbit IgG secondary antibody (1 : 100 dilution in blocking solution), as appropriate, at room temperature for 1 h. Coverslips were washed in PBS, excess moisture was removed with tissue and mounted on glass slides using Vectashield (Vector Laboratories) hard set mounting medium with DAPI. Immunofluorescence was visualised using a Nikon (Surrey, UK) E600 epifluorescence microscope.

## Results

### Clinicopathological details

The cohort consisted of 140 breast cancer patients with ER^−^ tumours. The median age was 55 years (IQR 45–64). In all, 94% of the cancer specimens were pathologically categorised as Grade 2 and 3, and the median size of the invasive cancer was 22 mm (IQR 15–30 mm). About 50% of the patients were axillary lymph node positive. At last follow-up, 41 patients had died of their disease, 18 of intercurrent disease while 75 patients were alive. The latter group had a mean patient follow-up of 13.1 years (minimum follow-up was 11.8 years and the maximum follow-up was 14.75 years). During the follow-up period, 47 patients experienced recurrence with a mean time of 4.9 years (minimum time to recurrence 0.5 years and maximum time to recurrence 8.5 years). The association with clinical parameters, recurrence (disease-free survival) and disease-specific survival are provided in Tables 1 and 2, respectively, and correlations between the clinicopathological characteristics of this cohort are shown in Table 3.

### *Sphingosine kinase 1 expression in ER^−^ breast cancer*

**Figure 1:**
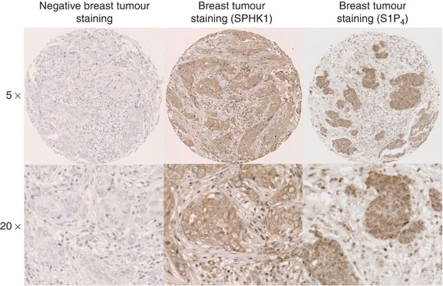
An example of SK1 and S1P_4_ expression detected in ER^−^ breast cancer samples with anti-SK1 or anti-S1P_4_ antibody, respectively.

Sphingosine kinase 1 expression was successfully assessed in all tumours analysed. A typical IHC using anti-SK1 antibody is shown in Figure 1. Antibody specificity for SK1 in IHC has previously been confirmed by us (Long *et al*, 2010a; Watson *et al*, 2010). Tumours were subdivided into those with high or low SK1 expression using the method described by Ruckäberle *et al* (2008). Therefore, low SK1 membrane expression was below 88 histoscore units (128 patients), low SK1 cytoplasmic expression was below 82 histoscore units (116 patients) and low SK1 nuclear expression was below 75 histoscore units (110 patients). *χ*^2^ analysis demonstrated that the membrane and cytoplasmic SK1 expression correlated with age. However, no correlations were observed between any other clinicopathological parameters or S1P_4_ levels (Table 3). Sphingosine kinase 1 expression at any cellular location was not associated with disease-free survival (Table 1) or disease-specific survival on univariate analysis (Table 2). However, high SK1 expression in the HER2^+^ tumours was significantly associated with shorter disease-specific survival (*P*=0.0001) compared with HER2^+^ patients with low SK1 expression in their tumours. Those patients with HER2-positive tumours and high SK1 expression have a mean disease-free survival of 2.9 years and a mean disease-specific survival time of 4.1 years compared with 6.45 years for disease-free survival and 12 years for disease-specific survival in the low tumour SK1 expression group. These results should be interpreted with some caution because of low patient numbers.

### *Sphingosine 1-phosphate expression in ER^−^ breast cancer*

**Figure 2:**
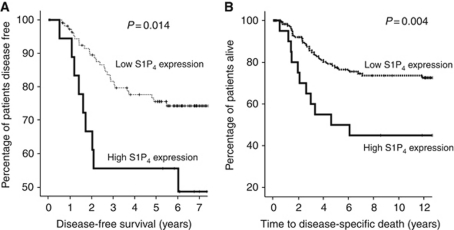
(**A**) High cytoplasmic S1P_4_ expression is associated with shorter disease-free survival. (**B**) High cytoplasmic S1P_4_ expression is associated with shorter disease-specific survival.

A typical IHC using anti-S1P_4_ antibody is shown in Figure 1. Antibody specificity for S1P_4_ has been previously confirmed by us (Long *et al*, 2010b). Sphingosine 1-phosphate receptor 4 expression was successfully assessed in all tumours analysed. Tumours were subdivided into those with high or low S1P_4_ expression using the method described by Ruckäberle *et al* (2008). Therefore, low S1P_4_ membrane expression was below 83 histoscore units (114 patients), low S1P_4_ cytoplasmic expression was below 82 histoscore units (120 patients) and low S1P_4_ nuclear expression was below 84 histoscore units (118 patients). *χ*^2^ analysis demonstrated that S1P_4_ expression was not correlated with clinicopathological parameters. However, the membrane, cytoplasmic and nuclear S1P_4_ expression levels correlated with each other (Table 3). High cytoplasmic tumour S1P_4_ (which might represent post-activated internalised receptor) expression in patients was associated with shorter disease-free survival (*P*=0.014. Table 1, Figure 2A) and disease-specific survival (*P*=0.004, Table 2, Figure 2B) compared with patients with low cytoplasmic tumour S1P_4_ expression on univariate analysis. Those patients with tumours that had high S1P_4_ expression have a mean disease-free survival of 5.2 years and a mean disease-specific survival time of 8 years compared with the low tumour S1P_4_ expression group, the latter having a disease-free survival time of 6.65 years and a mean disease-specific survival time of 11.7 years. When entered into multivariate analysis both disease-free survival and disease-specific survival were demonstrated to be independent of other significant clinicopathological parameters (Tables 1 and 2).

**Figure 3:**
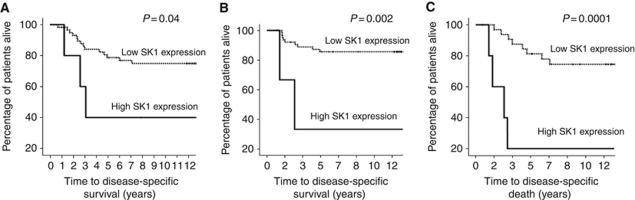
(**A**) High cytoplasmic SK1 expression in a low S1P_4_ expression background is associated with shorter disease-free survival compared with patients with low tumour S1P_4_ and SK1 expression. (**B**) High cytoplasmic SK1 expression in a low S1P_4_ expression background is associated with shorter disease-specific survival compared with patients with low tumour S1P_4_ and SK1 expression. (**C**) High SK1 or S1P_4_ expression is associated with shorter disease-specific survival compared with patients with low SK1 and S1P_4_ expression in their tumours.

Sphingosine 1-phosphate receptor 4 tumour expression was not associated with any one clinical subtype. However, high SK1 expression in tumours that also exhibited a low level of S1P_4_ expression was significantly associated with shorter disease-free survival and disease-specific survival (*P*=0.043 and *P*=0.033, respectively) compared with patients with low SK1 and low S1P_4_ tumour expression (Figures 3A and B). In addition, there was a significant association of shorter disease-specific survival in patients with tumours that express high SK1 or S1P_4_ (29 patients) compared with those that express low SK1 and S1P_4_ levels (108 patients; *P*=0.001; Figure 3C). Thus, patients whose tumours contain low levels of both SK1 and S1P_4_ have a mean disease-specific survival of 11.9 years compared with 7.95 years in patients with tumours that contain high SK1 or S1P_4_ expression (Figure 3C). This was independent of nodal status on multivariate analysis (*P*=0.016, hazard ratio of 2.3 (95% CI 1.2–4.412).

### *Functional interaction between S1P_4_, SK1 and HER2*

**Figure 4:**
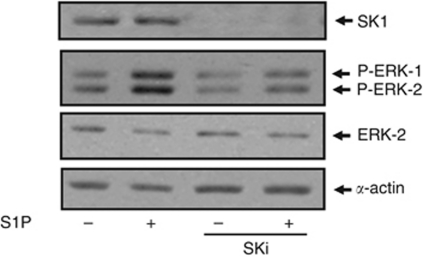
The effect of SKi on the ERK-1/2 pathway in MDA-MB-453 cells. MDA-MB-453 cells were treated with SKi (10 *μ*ℳ) for 24 h before stimulation with and without S1P (10 *μ*ℳ, 10 min). Western blots showing the effect of SKi on SK1 expression and the basal and S1P-induced activation of ERK-1/2 activation. Phosphorylated ERK-1/2 was detected with anti-phospho ERK-1/2 antibody and SK1 was detected with anti-SK1 antibody. ERK2 and *α*-actin was also detected to ensure comparable protein loading. Results are representative of three independent experiments.

**Figure 5:**
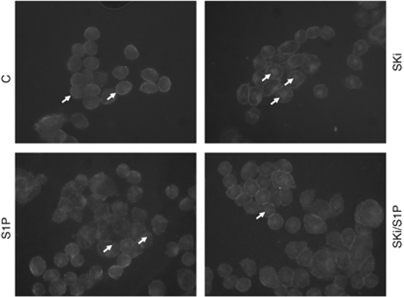
The effect of SKi on HER2 trafficking in MDA-MB-453 cells. MDA-MB-453 cells were treated with SKi (10 *μ*ℳ) for 15 min before stimulation with and without S1P (5 *μ*ℳ, 10 min). The images are immunofluorescence stains with anti-HER2 antibody showing the effect of SKi and/or S1P on the subcellular localisation of HER2. Results are representative of two experiments. The arrows in the control panel (C) indicate localisation of HER2 to the punctuate bodies at the plasma membrane, while in Ski- and S1P/Ski-treated cells they identify the localisation of HER2 to an intracellular compartment and small punctuate intracellular bodies. In the S1P panel, arrows identify HER2 localisation to small punctuate intracellular bodies.

We have previously demonstrated that the S1P-induced activation of ERK-1/2 in MDA-MB-453 cells involves S1P_4_ and HER2 (Long *et al*, 2010b). Thus, S1P stimulation of the ERK-1/2 pathway was reduced by siRNA knockdown of S1P_4_ or HER2, and by pharmacological inhibitors, including the S1P_2/4_ antagonist, JTE-013 and ErbB2 inhibitor II (Long *et al*, 2010b). Our finding that high SK1 expression in tumours that also contain low levels of S1P_4_ exhibit significantly shorter disease-free survival and disease-specific survival compared with patients with low SK1 and S1P_4_ tumour expression suggests that a functional interaction between SK1 and S1P_4_ might operate in ER^−^ breast cancer. To test this possibility *in vitro*, we assessed the effect of SK1 inhibitors on S1P_4_-mediated signalling in ER^−^ MDA-MB-453 cells. For this purpose, we used the SK1 inhibitors, SKi (2-(p-hydroxyanilino)-4-(p-chlorophenyl)thiazole)), which is a inhibitor of SK1 activity (Loveridge *et al*, 2010; Tonelli *et al*, 2010; Lim *et al*, 2011). We demonstrate here that the chronic treatment (24 h) of MDA-MB-453 cells with SKi promoted the loss of SK1 (Mr∼42 kDa) expression from these cells (Figure 4) consistent with our previous findings that SKi induces the ubiquitin-proteasomal degradation of SK1 in cancer cells (Loveridge *et al*, 2010; Tonelli *et al*, 2010; Lim *et al*, 2011). SKi also induced a substantial reduction in S1P-stimulated activation of ERK-1/2 (Figure 5), thereby providing evidence for the existence of a functional S1P_4_/SK1 regulatory pathway regulating ERK-1/2 in these cells. As HER2 is essential for S1P stimulation of ERK-1/2 (Long *et al*, 2010b), the current data also define a functional interaction between SK1 and HER2. In addition, we have previously shown that basal ERK-1/2 activation is dependent on HER2 tyrosine kinase activity and is independent of S1P_4_ (Long *et al*, 2010b). It is therefore noteworthy that the treatment of MDA-MB-453 cells with SKi reduced basal ERK-1/2 activation (Figure 4). We have also found that acute treatment (15 min) of MDA-MB-453 cells with SKi altered HER2 trafficking in MDA-MB-453 cells (Figure 5). Immunofluorescence staining of unstimulated MDA-MB-453 cells with anti-HER2 antibody demonstrates that HER2 is localised in punctuate bodies at the plasma membrane/cell periphery (Figure 5). Treatment of these cells with SKi causes a marked redistribution of HER2, which localised into cytoplasmic punctuate bodies and accumulated into an unidentified intracellular compartment (Figure 5). In contrast, the treatment of these cells with S1P induced the re-localisation of HER2 (in punctuate bodies) from the plasma membrane to the cytoplasm, with little if any accumulation into the intracellular compartment (Figure 5).

## Discussion

The major finding of this study is that S1P_4_ is linked with poor prognosis in ER^−^ breast cancer patients as evidenced by shorter disease-free survival and disease-specific survival of patients who have high S1P_4_ expression in their tumours compared with those that have low receptor expression. These findings are significant because S1P_4_ has a restricted tissue distribution, being localised largely to immune cells. The receptor is therefore a possible target for drug intervention because of the potential to limit side effects. More importantly, we have previously demonstrated a functional coupling of S1P_4_ with HER2, suggesting that combined treatment of cells with S1P_4_ antagonists and ErbB2 inhibitors might represent a more effective treatment regime for ER^−^ breast cancer patients compared with ErbB2 inhibitors alone.

We also demonstrate here that while SK1 expression was not associated with disease-free survival or disease-specific survival in aggressive tumours when considered alone, high SK1 expression was associated with shorter disease-free and disease-specific survival in patients whose tumour contained low levels of S1P_4_. These findings demonstrate that SK1 might become more important when combined with S1P_4_, suggesting a functional link between these two proteins that is defined by their expression levels. Further evidence to support this functional interaction was obtained by the demonstration that the SK1 inhibitor, SKi, reduced SK1 expression and decreased the S1P/S1P_4_-induced stimulation of ERK-1/2 in MDA-MB-453 cells. There are several possibilities for how SK1 and S1P_4_ might interact functionally. For instance, ‘inside-out’ signalling involves activation of SK1 and the subsequent release of S1P from cells, which might then act on S1P_4_ receptors on the cancer cell. Alternatively, S1P binding to S1P_4_ might induce activation of SK1, and the resulting S1P formed might function to activate intracellular proteins to promote cancer cell growth/survival. Therefore, stimulation of S1P_4_ at low expression might be increased by S1P formed from highly expressed SK1 (e.g., inside-out signalling). Alternatively, signalling from S1P_4_ might be amplified by downstream activation of highly expressed SK1 in the tumours. Moreover, we have previously demonstrated that exogenous S1P (via S1P_3_) induces the translocation of SK1 from the cytoplasm to the plasma membrane of ER^+^ MCF-7 cells, and that siRNA knockdown of SK1 in these cells reduces the activation of ERK-1/2 by exogenous S1P (Long *et al*, 2010a).

We have also reported here that high SK1 expression in the ER^−^/HER2^+^ tumours is significantly associated with shorter disease-specific survival compared with HER2^+^ patients with low SK1 expression in their tumours. A functional interaction between HER2 and SK1 in MDA-MB-453 cells is evidenced by the demonstration that pharmacological inhibition of SK1 activity results in altered HER2 trafficking in MDA-MB-453 cells. The interaction of SK1 and HER2 suggests that their cooperation enhances ER^−^ cancer progression. This contrasts with the functional interaction between SK1 and HER2 in ER^+^ breast cancer patients (Long *et al*, 2010a). In this regard, we have demonstrated that HER2 increases SK1 expression in ER^+^/HER2^+^ breast cancer cells (Long *et al*, 2010a). This leads to a negative feedback loop in which SK1 induces a reduction in HER2 expression and ablates S1P-stimulated migration; the latter due to an SK1-induced deactivation/degradation of p21 activated protein kinase 1, which is normally required for motility. Moreover, we have shown that high SK1 expression in the ER^+^/HER2^+^ breast cancer tumours is correlated with increased patient survival and reduced disease recurrence on Tamoxifen (Long *et al*, 2010a), thereby demonstrating a protective role for SK1 in this tumour phenotype (Long *et al*, 2010a).

Taken together, these findings highlight the different effect of S1P signalling signatures in ER^+^ and ER^−^ breast cancers on clinical prognosis, influenced by S1P receptor subtypes, SK1 and HER2. The findings in this study also highlight the need to define the role of S1P receptor subtypes, SK1 and HER2 in shaping the precise cancer disease phenotype in order to inform on the best therapeutic approach. Our findings suggest that both S1P_4_ and SK1 represent novel biomarkers predictive of prognostic significance in ER^−^ breast cancer. Indeed, the S1P_4_-SK1 regulatory module might represent an important target for drug intervention designed to prevent ER^−^ breast cancer progression.

## Figures and Tables

**Table 1:** The patient cohort’s characteristics correlated with disease-free survival

		**Univariate**	**Multivariate**		
**Patient cohort 140**	**Numbers**	***P*** **-value**	***P*** **-value**	**HR**	**IQR**
Age (<50 year/>50 year)	48/92	0.639			
Tumour type (duct/lob/tub/others)	129/2/9	0.190			
Grade (unknown/G1/G2/G3)	4/4/25/107	0.492			
Size (mm; unknown/<20, 20–50, >50)	4/64/69/2	<0.001	NS		
Lymph node (unknown/positive/negative)	1/70/67	0.020	0.045	2.1	1.1–4.2
HER2 status (unknown/positive/negative)	1/100/39	0.497			
					
Ki67 (unknown/low/ moderate/high)	2/73/43/22	0.088			
					
SK1 membrane (positive/negative)	128/12	0.121			
SK1 cytoplasmic (positive/negative)	116/24	0.276			
SK1 nuclear (positive/negative)	110/30	0.595			
S1P_4_ membrane (positive/negative)	114/26	0.557			
S1P_4_ cytoplasmic (positive/negative)	120/20	0.014	0.026	2.4	1.1–5.2
S1P_4_ nuclear (positive/negative)	118/22	0.214			

Abbreviations: HER2=human epidermal growth factor receptor 2; HR=hazard ratio; IQR=interquartile range; NS=non significant; S1P_4_=sphingosine 1-phosphate receptor 4; SK1=sphingosine kinase 1.

Each clinical and pathological parameter was correlated to recurrence (*P*-values). Grade=Bloom and Richardson grade; Histology: duct=ductal carcinoma; lob=lobular carcinoma; tub=tubular carcinoma; others including mucinous, mucoid and micropapillary carcinoma. Univariate analysis is performed for each parameter. However, only markers with a *P*-value of <0.05 are included in the multivariate model.

**Table 2:** The patient cohort’s characteristics correlated to disease-specific survival

		**Univariate**	**Multivariate**		
**Patient cohort 140**	**Numbers**	***P*** **-value**	***P*** **-value**	**HR**	**IQR**
Age (<50 year/>50 year)	48/92	0.941			
Tumour type (duct/lob/tub/others)	129/2/9	0.142			
Grade (unkown/G1/G2/G3)	4/4/25/107	0.444			
Size (mm; unkown/<20, 20–50, >50)	4/64/69/2	0.088			
Lymph node (unkown/positive/negative)	1/70/67	0.001	0.002	3.0	1.5–6.0
HER2 status (unkown/positive/negative)	1/100/39	0.806			
					
Ki67 (unknown/low/ moderate/high)	2/73/43/22	0.109			
					
SK1 membrane (positive/negative)	128/12	0.118			
SK1 cytoplasmic (positive/negative)	116/24	0.559			
SK1 nuclear (positive/negative)	110/30	0.563			
S1P_4_ membrane (positive/negative)	114/26	0.539			
S1P_4_ cytoplasmic (positive/negative)	120/20	0.004	0.031	2.27	1.1–4.5
S1P_4_ nuclear (positive/negative)	118/22	0.392			

Abbreviations: HER2=human epidermal growth factor receptor 2; HR=hazard ratio; IQR=interquartile range; S1P_4_=sphingosine 1-phosphate receptor 4 ; SK1=sphingosine kinase 1.

Each clinical and pathological parameter was correlated to disease-specific survival (*P*-values). Grade=Bloom and Richardson grade; Histology: duct=ductal carcinoma; lob=lobular carcinoma; tub=tubular carcinoma; others including mucinous, mucoid and micropapillary carcinoma. Univariate analysis is performed for each parameter. However, only markers with a *P*-value of <0.05 are included in the multivariate model.

**Table 3:** Correlations between SK1 and S1P_4_, expression and the clinicopathological characteristics of the cohort

**Variables**	**Tumour type**	**Grade**	**Size**	**LN status**	**HER status**	**Ki67 status**	**SK1 mem**	**SK1 cyto**	**SK1 nuc**	**S1P**_**4**_**mem**	**S1P**_**4**_**cyto**	**S1P**_**4**_**nuc**
Age (<50 year/>50 year)	NS	NS	NS	NS	NS	NS	NS	0.025	0.029	NS	NS	NS
Tumour type (duct/lob/tub/others)		<0.001	NS	NS	NS	NS	NS	NS	NS	NS	NS	NS
Grade (G1/G2/G3)			0.024	0.001	NS	0.05	NS	NS	NS	NS	NS	NS
Size (mm; <20, 20–50, >50)				0.013	NS	NS	NS	NS	NS	NS	NS	NS
Lymph node (positive/negative)					NS	NS	NS	NS	NS	NS	NS	NS
HER2 status (positive/negative)						0.037	NS	NS	NS	NS	NS	NS
Ki67 status (low/moderate/high)							NS	NS	NS	NS	NS	NS
												
SK1 membrane (positive/negative)								NS	NS	NS	NS	NS
SK1 cytoplasmic (positive/negative)									NS	NS	NS	NS
SK1 nuclear (positive/negative)										NS	NS	NS
S1P_4_ membrane (positive/negative)											<0.001	<0.001
S1P_4_ cytoplasmic (positive/negative)												0.011

Abbreviations: cyto=cytoplasmic; HER2=human epidermal growth factor receptor 2; HR=hazard ratio; IQR=interquartile range; mem=membrane; nuc=nuclei; NS=non significant; IL=lymph node; S1P_4_=sphingosine 1-phosphate receptor 4; SK1=sphingosine kinase 1.

Tumour type: duct=ductal carcinoma; lob=lobular carcinoma; tub=tubular carcinoma; others including mucinous, mucoid and micropapillary carcinoma; Grade: Bloom and Richardson grade. *χ*^2^ test: NS *P*-values.
