# Expression of sphingosine 1-phosphate receptor 4 and sphingosine kinase 1 is associated with outcome in oestrogen receptor negative breast cancer

**DOI:** 10.1038/bjc.2012.352

**Published:** 2012-08-07

**Authors:** J Ohotski, J S Long, C Orange, B Elsberger, E Mallon, J Doughty, S Pyne, N J Pyne, J Edwards

**Correction to**: *British Journal of Cancer* (2012) **106**, 1453–1459; doi:10.1038/bjc.2012.98

In revision of the above paper during the proofing and correction process, an earlier version of Figure 3 was resupplied for publication and, subsequently, published in error. The authors and publishers apologise for this mistake.

The correct Figure 3 and associated legend are shown below.

## Figures and Tables

**Figure 3:**
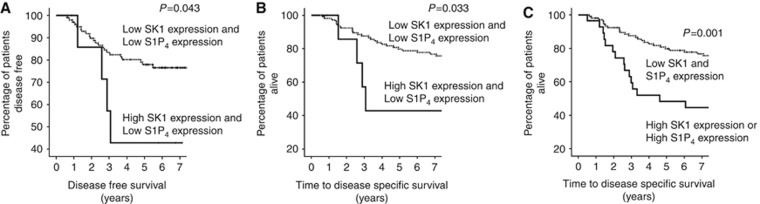
(**A**) High cytoplasmic SK1 expression in a low S1P_4_ expression background is associated with shorter disease-free survival compared with patients with low tumour S1P_4_ and SK1 expression. (**B**) High cytoplasmic SK1 expression in a low S1P_4_ expression background is associated with shorter disease-specific survival compared with patients with low tumour S1P_4_ and SK1 expression. (**C**) High SK1 or S1P_4_ expression is associated with shorter disease-specific survival compared with patients with low SK1 and S1P_4_ expression in their tumours.

